# Pneumorrhachis of Infectious Origin in a Patient with Advanced HIV and Uncontrolled Diabetes: A Case Report and Review of the Literature

**DOI:** 10.7759/cureus.102490

**Published:** 2026-01-28

**Authors:** Thenell Van der Westhuizen, Abdullah E Laher

**Affiliations:** 1 Emergency Medicine, Charlotte Maxeke Johannesburg Academic Hospital, Johannesburg, ZAF

**Keywords:** diabetic ketoacidosis, epidural air, hiv, mrsa infection, pneumorrhachis

## Abstract

Pneumorrhachis, the presence of air within the spinal canal, is an uncommon radiological finding, typically associated with trauma or pneumomediastinum. Non-traumatic causes, particularly infective ones, are exceedingly rare. We describe a woman with advanced HIV infection and poorly controlled diabetes mellitus who presented with confusion and meningism in the context of diabetic ketoacidosis (DKA). Computed tomography (CT) revealed cervical extradural pneumorrhachis. Blood, urine, sputum, and cerebrospinal fluid (CSF) cultures confirmed disseminated methicillin-resistant *Staphylococcus aureus* (MRSA) infection. This case adds to the limited literature describing pneumorrhachis of infectious origin unrelated to trauma or pneumomediastinum. A review of published cases shows that most are associated with emphysematous infections (epidural abscesses, pyelonephritis, cystitis, or meningitis) caused by gas-forming organisms. While pneumorrhachis itself is often incidental and self-limiting, it is an indicator of severe underlying disease. However, due to the limited number of reported cases, morbidity and mortality are yet to be defined. Recognition of pneumorrhachis should prompt urgent investigation for an infective source and initiation of appropriate antimicrobial therapy.

## Introduction

Pneumorrhachis, defined as the presence of air within the spinal canal, is an exceptional radiological finding first described in 1977 [1]. The condition is broadly classified as traumatic or non-traumatic. Non-traumatic pneumorrhachis may arise spontaneously or from iatrogenic, infectious, or neoplastic processes. In most cases, air reaches the spinal canal secondarily, typically via pneumomediastinum, infected sacral ulcers, or invasive procedures such as lumbar puncture. In the case of infectious pneumorrhachis, air created by gas-forming organisms enters the spinal canal via direct extension, dural breach, or inflammatory dissection of tissue between the source and the spinal canal. All of these cases involved patients with immunocompromised or epidural abscesses [1,2]. Diagnosis is radiological. It is often an incidental finding when imaging is requested for another concern, such as delirium, pyelonephritis, or epidural abscess. CT of the spine is the most sensitive modality, whereas MRI is useful when concomitant pathology (e.g., abscess or tumor) is suspected. Plain radiographs are rarely diagnostic but may help identify pneumomediastinum as a potential source. Once identified, investigations should aim to determine the underlying cause, for instance, chest radiography for pneumomediastinum, urinalysis for urinary tract infections, or cerebrospinal fluid (CSF) analysis for meningitis. Management should be individualized. Most patients can be treated conservatively through management of the underlying condition, supplemental oxygen, and supportive care. Surgical decompression is reserved for rare cases complicated by neurological deficit or cord compression. While pneumorrhachis itself is self-limiting, it is an indicator of severe underlying disease. The following case highlights an infective cause of pneumorrhachis secondary to disseminated methicillin-resistant *Staphylococcus aureus* (MRSA) infection. It also illustrates the severity and rapid disease progression often associated with pneumorrhachis.

## Case presentation

**Figure 1:**
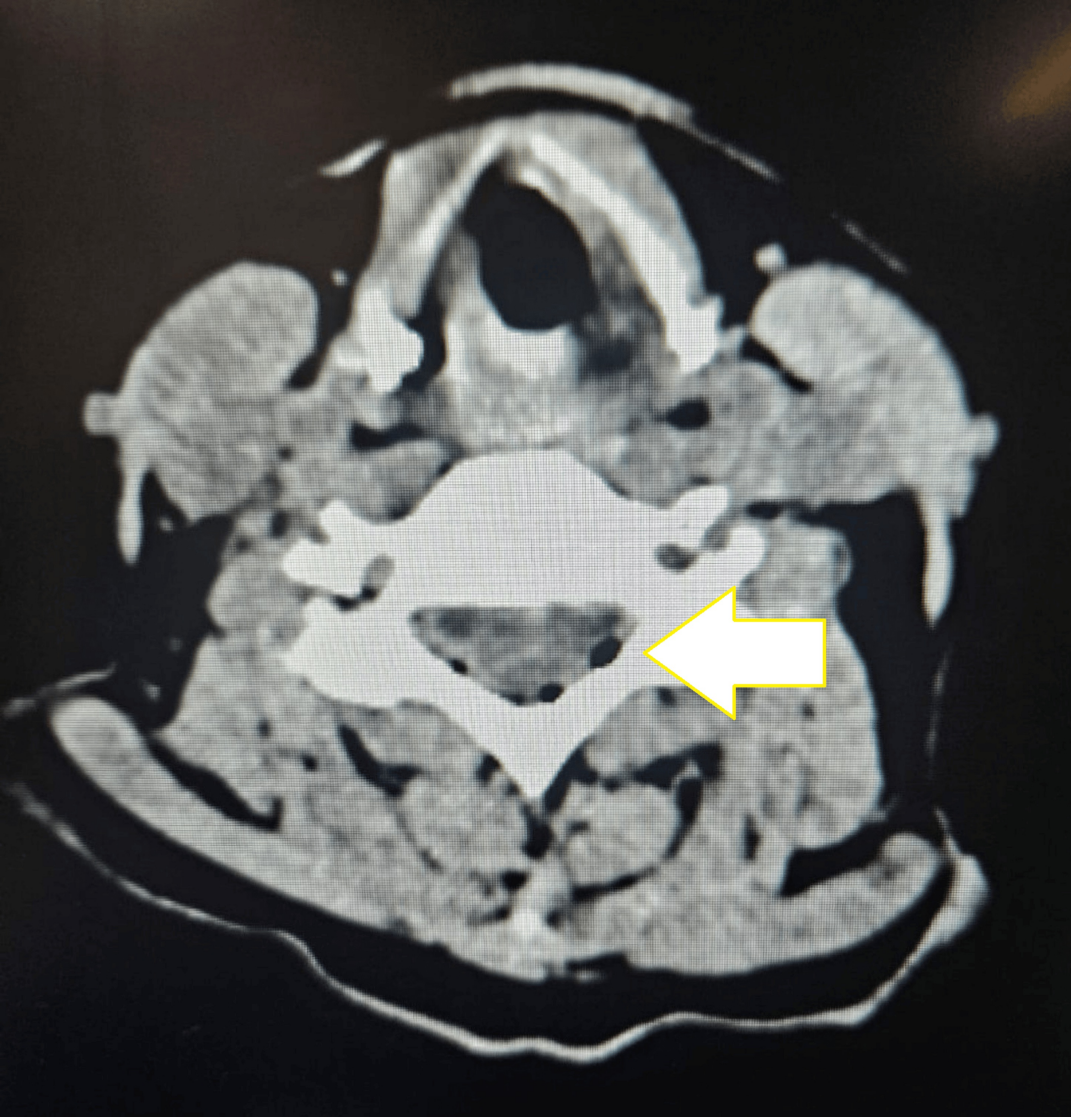
Non-contrast CT of the brain (axial plane) showing cervical extradural pneumorrhachis (C1–C4) with associated retropharyngeal and paravertebral air locules. Pneumorrhacis is indicated by the white arrow.

A woman in her mid-fifties presented to the emergency department with a two-week history of confusion and generalized weakness. She had known HIV infection and poorly controlled diabetes mellitus. Her CD4 count was 89, and HbA1c was 15.4%. On examination, she appeared acutely ill, with a Glasgow Coma Scale score of 11 and marked neck stiffness. There was no history or clinical evidence of trauma, sacral ulcers, or prior lumbar puncture. Initial investigations demonstrated sepsis complicated by diabetic ketoacidosis (DKA) and acute kidney injury. A non-contrast CT of the brain was performed because of her altered level of consciousness. The scan revealed pneumorrhachis with gas locules in the extradural spinal canal extending from C1 to C4 (Figure 1). Additional small air locules were seen in the cervical paravertebral space, with minimal fat stranding but no muscular edema or fluid collections to suggest an abscess. Scant air was also seen in the retropharyngeal space without associated swelling or effusion. A chest radiograph excluded pneumothorax or pneumomediastinum as a possible source.

Lumbar puncture revealed elevated protein and pleocytosis with both polymorphonuclear and lymphocytic predominance. Blood, urine, sputum, and CSF cultures all grew MRSA, confirming disseminated MRSA infection. The patient was resuscitated with intravenous fluids, started on ceftriaxone, and treated for DKA in the emergency department. Blood, CSF, and urine cultures performed on day 1 of presentation indicated MRSA and vancomycin sensitivity. Antibiotic therapy was changed from ceftriaxone to intravenous vancomycin on day 2. The patient was given a fluid preload with each vancomycin dose, and urea and creatinine were monitored daily. Despite appropriate fluids and antibiotic therapy, she developed progressive renal failure and nosocomial pneumonia. Blood results showed an estimated glomerular filtration rate that diminished from normal to 10 ml/min/1.73 m², procalcitonin that climbed from 0.4 to 1.79 ng/ml, and C-reactive protein that continued to increase despite culture-directed antibiotics. On day 10 in the medical ward, she was intubated and ventilated for worsening encephalopathy. A repeat CT demonstrated resolution of the pneumorrhachis with no new findings. Unfortunately, she died from disseminated MRSA sepsis on day 11 of hospitalization.

## Discussion

The true incidence of pneumorrhachis remains unknown due to its rarity and diverse etiologies. However, reported cases have increased in recent years, likely reflecting advances in imaging and greater awareness among clinicians. Infectious pneumorrhachis is exceptionally uncommon. Signs and symptoms are mainly related to the underlying infection; however, the patient may complain of back pain or neck stiffness. Due to the vagueness of signs and symptoms, the diagnosis is exclusively made on imaging while investigating the underlying infection. Air may be introduced into the spinal canal through several mechanisms: direct extension from adjacent emphysematous infections such as osteomyelitis, pyelonephritis, or cystitis; meningitis due to gas-forming organisms; or fistulous tracts from infected pressure ulcers or malignancy, resulting in CSF leakage and negative-pressure air entry. The most frequently reported organisms include *Escherichia coli*, *Klebsiella *spp., *Clostridium* spp., *Staphylococcus aureus*, *Streptococcus *spp., *Citrobacter *spp., *Morganella *spp., and other enteric pathogens. In this case, disseminated MRSA infection was implicated, a rare finding. A summary of previously published cases is presented in Table 1. A systematic narrative review was performed to identify published cases and studies describing pneumorrhachis of infectious origin. PubMed/Medical Literature Analysis and Retrieval System Online (MEDLINE) and Scopus were searched from inception to the date of manuscript preparation.

**Table 1:** Published cases of infectious (non-traumatic) pneumorrhachis

Author	Date of Publication	Gender	Age (Years)	Risk Factors	Symptoms	Outcome	Pathogen
Kirzner et al. [3]	1988	F	68	Diabetes mellitus	Epidural abscess, back pain, bilateral leg paralysis, fever	Completely recovered	Staphylococcus aureus
Shintani et al. [4]	1992	F	71	Contaminated epidural catheter	Epidural abscess, headache, fever	Completely recovered	Staphylococcus aureus
Kökes et al. [5]	1993	M	52	Diabetes mellitus	Epidural abscess, back pain, paraparesis	Demised secondary to septic shock	Streptococcus, Peptostreptococcus, Bacteroides
Fujisawa et al. [6]	1998				Epidural abscess, neck pain, fever, paraparesis	Completely recovered	Staphylococcus aureus
Nakatani et al. [7]	1998	F	54	Nil	Epidural abscess, fever, decubitus ulcer	Paralysis of both legs	Bacteroides fragilis, Peptostreptococcus
Jomir et al. [8]	2009	M	27	Paraplegia	Sacral pressure ulcer, orthostatic headache, and nausea	Discharged	*Staphylococcus aureus*, Group B Streptococcus
Hur et al. [9]	2012	M	68	Anterior cervical surgery	Epidural abscess, neck pain, paraparesis, urinary retention	Died secondary to septic shock	Streptococcus anginosus
Hur et al. [9]	2012	M	52	Anterior cervical surgery	Neck pain, epidural abscess	Discharged	Methicillin-resistant *Staphylococcus aureus*
Lee et al. [10]	2013	M	72	Vertebroplasty	Paralysis of both legs, back pain	Completely recovered	Aeromonas hydrophila
Amara et al. [11]	2013	M	21		Headache, fever, vomiting, dyspnea	Died	Bacterial meningitis (unspecified)
Akagawa et al. [12]	2015	F	78	Diabetes mellitus	Epidural abscess, back pain, paralysis of the legs	Completely recovered	Clostridium perfringens, Escherichia coli, Enterococcus faecalis
Kim and Kim [13]	2017	M	56	Nil	Epidural abscess, neck pain, fever	Paralysis and urinary incontinence	Streptococcus anginosus
Matsuo et al. [14]	2019	F	70	Diabetes mellitus	Epidural abscess, back pain, left leg paralysis	Completely recovered	Escherichia coli
Miranda et al. [15]	2021	F	67	Diabetes mellitus, Hypertension, Alzheimer’s, bedbound	Sacral decubitus ulcer, confusion	Died	Streptococcus anginosus
Navriya et al. [16]	2021	M	65	Diabetes mellitus	Emphysematous pyelonephritis, flank pain, fever, hypotension	Completely recovered	Escherichia coli
Maegawa et al. [17]	2022	M	68	Parkinson’s disease, bedbound	Fever	Discharged	Clostridium perfringens
Ehret et al. [18]	2022	M	88	Diabetes mellitus, Crohn’s disease with chronic steroid use	Back pain and confusion, emphysematous cystitis	Discharged to hospice	Escherichia coli
Sumitro et al. [19]	2023	M	57	Hepatitis C	Fever, back pain, weakness, dysuria, prostate abscess	Discharged	Klebsiella pneumoniae
Allena et al. [20]	2023	F	77	Diabetes mellitus, Hypertension	Emphysematous cystitis, paraspinal abscess, leg weakness	Discharged to hospice	Klebsiella pneumoniae

Of reported cases, the average age was 61.7 years, 57.9% of patients were male (11 males and eight females). The most common comorbidity was diabetes mellitus, while four patients presented after an invasive procedure. Epidural abscess was present in 52.6% of reported cases. The most common symptoms reported were back or neck pain (57.9%), fever (47.4%), and leg paraparesis or paralysis (42%). Outcomes varied: 36.8% completely recovered, while 21% of patients died.

A review of reported cases demonstrates that air enters the spinal canal via contiguous spread from adjacent emphysematous infections, gas-forming meningitis, or secondary to invasive procedures. The microbiological spectrum is broad and predominantly involves gas-forming or enteric organisms, while *Staphylococcus aureus*, and especially MRSA, remains an infrequent cause. Outcomes vary widely, ranging from complete neurological recovery to death from overwhelming sepsis, highlighting the importance of early recognition, prompt imaging, and aggressive management of the underlying cause.

## Conclusions

Non-traumatic pneumorrhachis is a rare but clinically significant entity. When identified, it should trigger an urgent search for a possible infective source, particularly in immunocompromised patients. As demonstrated by this case, though the intraspinal air may resolve spontaneously, its presence often signifies severe underlying disease. Early recognition, diagnosis, and aggressive management of sepsis are essential for improving outcomes. Further research is needed to clarify optimal management strategies and long-term prognosis for non-traumatic, infection-related pneumorrhachis.
